# Development and validation of a novel signature to predict the survival and affect the immune microenvironment of esophageal squamous cell carcinoma: epigenetic-related genes

**DOI:** 10.3389/fimmu.2025.1670600

**Published:** 2025-10-23

**Authors:** Yani Su, Ming Zhang, Qiong Zhang, Pengfei Wen, Ke Xu, Jiale Xie, Xianjie Wan, Lin Liu, Peng Xu, Zhi Yang, Mingyi Yang

**Affiliations:** ^1^ Department of Radiotherapy, Tangdu Hospital, Fourth Military Medical University, Xi’an, Shaanxi, China; ^2^ Department of General Practice, Honghui Hospital, Xi’an Jiaotong University, Xi’an, Shaanxi, China; ^3^ Department of Joint Surgery, HongHui Hospital, Xi’an Jiaotong University, Xi’an, Shaanxi, China

**Keywords:** esophageal squamous cell carcinoma, epigenetic, prognosis, immune microenvironment, gene

## Abstract

**Objective:**

Esophageal squamous cell carcinoma (ESCC) is a malignancy characterized by extensive epigenetic dysregulation. This study aims to develop a robust prognostic model utilizing epigenetic-related genes (ERGs) to improve survival prediction in ESCC patients, while simultaneously elucidating potential mechanisms underlying immune microenvironment modulation.

**Methods:**

This study employed transcriptomic data from The Cancer Genome Atlas (TCGA) as the training cohort and data from GSE53625 in the Gene Expression Omnibus (GEO) as an independent validation cohort. A total of 796 epigenetic regulator genes (ERGs) were curated from the EpiFactors database and intersected with TCGA-ESCC gene expression profiles to identify ESCC-associated ERGs. Differential expression analysis was then conducted to identify differentially expressed ERGs (DE-ERGs). Using univariate Cox and LASSO regression analyses, a prognostic risk model was constructed and thoroughly evaluated through risk stratification curves, survival status distribution maps, risk score heatmaps, survival analysis, ROC curves, and multivariate Cox regression. Further analyses included assessing the prognostic model’s association with clinical features and risk stratification. To investigate the immune microenvironment, immune cell infiltration correlation, single-sample gene set enrichment analysis (ssGSEA), and immune checkpoint profiling were performed. Drug sensitivity analysis was also carried out to identify potential therapeutic agents showing differential efficacy between risk subgroups. Finally, the expression patterns of key prognostic ERGs were validated using RT-qPCR.

**Results:**

Through comprehensive differential expression analysis, we identified 345 DE-ERGs in ESCC. A robust prognostic signature comprising 13 critical ERGs—PIWIL4, SATB1, GSE1, NCOR1, BUB1, SAP30L, CHEK1, MASTL, ATM, BMI1, DNAJC2, UBE2D1, and SSRP1—was established using univariate Cox regression followed by LASSO penalized regression analysis. The prognostic efficacy of this signature was confirmed through multidimensional assessments using independent GEO datasets. Immunological characterization revealed significant enrichment of CD8^+^ T cells, DCs, and pDCs in high-risk patients, along with elevated cytolytic activity, HLA expression, and MHC class I activity. Additionally, three immune checkpoint molecules—TMIGD2, IDO1, and CD44—were found to be differentially expressed between risk groups. Drug sensitivity analysis identified four promising therapeutic compounds—PD-0325901, Bryostatin-1, ATRA, and Roscovitine—with potential clinical utility for ESCC treatment. Experimental validation via RT-qPCR confirmed consistent overexpression of GSE1, NCOR1, BUB1, CHEK1, UBE2D1, and SSRP1 in ESCC cell lines, whereas PIWIL4 and ATM showed significant downregulation.

**Conclusion:**

The findings of this study offer clinically relevant insights for prognostic stratification and characterization of the immune microenvironment in ESCC patients. Moreover, these results provide novel perspectives that may contribute to the development of more effective prognostic tools and targeted therapeutic strategies for ESCC management.

## 1 Introduction

Esophageal cancer (EC) is among the most aggressive malignancies, ranking as the seventh most commonly diagnosed cancer and the sixth leading cause of cancer-related mortality worldwide (1). Esophageal squamous cell carcinoma (ESCC) is the predominant histological subtype and continues to exhibit high incidence and mortality, especially in regions such as China (2, 3). Although advances in early diagnostic techniques and multimodal treatments have led to modest improvements in outcomes, the prognosis for ESCC patients remains poor, with a 5-year overall survival rate of only 15–20% (4). Recent research has increasingly focused on elucidating the molecular mechanisms of ESCC pathogenesis, with numerous studies seeking biomarkers to enhance risk stratification, guide therapy, and improve prognostic accuracy (5–7). However, the molecular drivers of ESCC are still not fully understood, and reliable biomarkers for early detection, monitoring progression, and predicting outcomes remain lacking. Due to its significant clinical burden and the limitations of current treatments, ESCC continues to represent a major public health challenge. Thus, there is a pressing need for innovative therapeutic approaches and better prognostic tools to reduce the global health impact of this devastating disease.

Cancer progression is driven by the accumulation of genomic alterations, including both genetic and epigenetic aberrations. While genetic mutations directly disrupt DNA sequences, epigenetic modifications—such as changes in DNA methylation and histone post-translational modifications—orchestrate tumorigenesis by dysregulating transcriptional programs that drive malignant transformation (8). These two key epigenetic mechanisms, DNA methylation and histone marking, play a pivotal role in tumor development, metastatic dissemination, and therapeutic resistance. Their cancer-specific patterns have become valuable biomarkers for diagnostic stratification, disease monitoring, and personalized treatment selection, thereby supporting improved clinical decision-making (8). Moreover, pervasive dysregulation of epigenetic processes is now recognized as a hallmark of cancer (9). In recent years, immunotherapy has revolutionized oncology with unprecedented breakthroughs in cancer treatment. Importantly, epigenetic profiles of both immune and tumor cells show significant potential as predictive biomarkers for patient response to immunotherapeutic interventions (10). Growing evidence indicates that tumors exploit diverse epigenetic mechanisms to evade immune surveillance, highlighting a critical interplay between epigenetics and antitumor immunity (11). Consequently, epigenetic-targeting agents have attracted considerable attention as potent immunomodulators, offering promising avenues for enhancing the efficacy of cancer immunotherapy (11).

**Figure 1:**
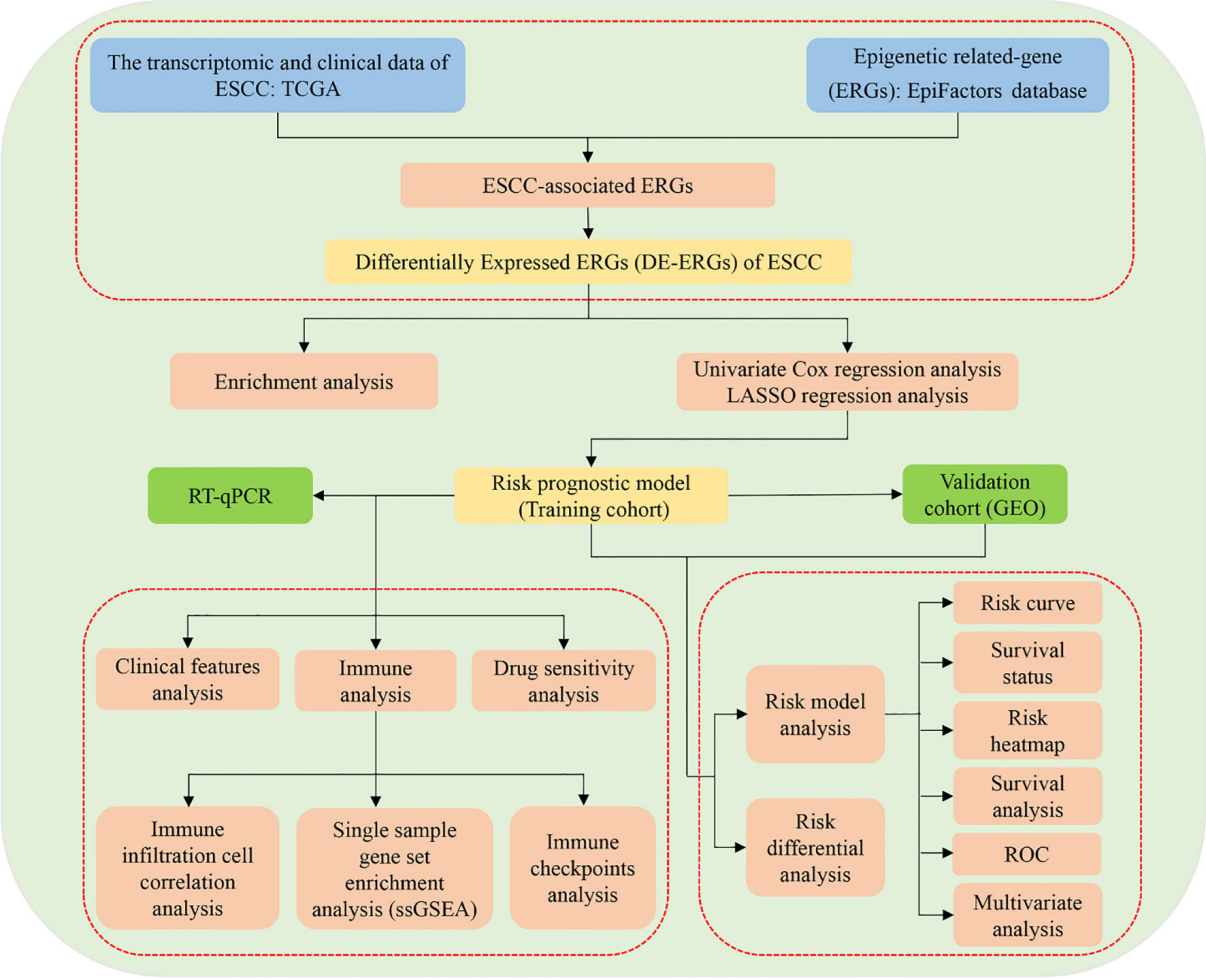
The flowchart of this study.

Emerging research has increasingly highlighted the pivotal role of epigenetic dysregulation in the pathogenesis of ESCC (12). For instance, the epigenetic regulator KDM4D has been identified as a tumor suppressor in ESCC, exerting its effects through modulation of the SYVN1/HMGB1 ubiquitination axis (12). Additionally, a positive feedback loop involving NKX2-5/LHX1 and UHRF1 has been implicated in ESCC tumorigenesis via epigenetic mechanisms (13), while JMJD3 contributes to malignant progression through epigenetic activation of the MYC oncogene (9). Recent studies have further identified novel epigenetic drivers, such as non-canonical WNT/β-catenin/MMP signaling activation and a YY1-mediated regulatory network involving the long non-coding RNA ESCCAL-1 and ribosomal proteins (14). Despite these advances, critical gaps remain in understanding how epigenetic modifications influence ESCC prognosis and shape the tumor immune microenvironment. The development of robust risk prediction models has emerged as a powerful tool for improving prognostic assessment and immuno-oncology research (15, 16). In this context, the present study aims to systematically investigate the impact of epigenetic alterations on ESCC prognosis and the immune landscape by constructing and validating a risk prediction model based on epigenetic-related genes (ERGs). The research design and analytical workflow are summarized in **Figure 1**.

## 2 Materials and methods

### 2.1 Data acquisition and collation

In this study, transcriptomic data from The Cancer Genome Atlas (TCGA) (https://portal.gdc.cancer.gov/) served as the primary training cohort, consisting of 81 ESCC specimens and 11 normal tissue samples. The TCGA dataset provided comprehensive clinicopathological annotations, including gender, age, TNM stage, and overall clinical stage. For independent validation, we used the GSE53625 dataset from the Gene Expression Omnibus (GEO) repository (https://www.ncbi.nlm.nih.gov/geo/), which comprises 179 paired tumor-normal samples along with clinical metadata such as age, T stage, N stage, and clinical stage. The epigenetic landscape was characterized using 796 ERGs obtained from the EpiFactors database (https://epifactors.autosome.org/), a comprehensive resource for epigenetic regulators.

### 2.2 Differentially expressed ERGs of ESCC

To identify epigenetically relevant genes in ESCC, we first intersected the TCGA-ESCC transcriptome dataset with 796 known ERGs from the EpiFactors database, obtaining a subset of ESCC-associated ERGs. Subsequently, differential expression analysis was performed on these candidate genes using stringent statistical criteria (P < 0.05 and |logFC| ≥ 1) with the limma package in R, identifying significantly dysregulated ERGs in ESCC compared to normal controls. This systematic approach enabled the robust identification of differentially expressed ERGs (DE-ERGs) implicated in ESCC pathogenesis.

### 2.3 Enrichment analysis

To elucidate the biological significance and pathway involvement of the DE-ERGs in ESCC, we conducted comprehensive functional enrichment analyses. Gene Ontology (GO) annotation was performed using the clusterProfiler package in R, while pathway enrichment analysis was carried out through Gene Set Enrichment Analysis (GSEA) to identify significantly altered pathways. A statistical significance threshold of P < 0.05 was applied to all enrichment analyses.

### 2.4 Construction of risk prognostic model

To identify prognostic DE-ERGs in ESCC, we first conducted univariate Cox proportional hazards regression analysis (P < 0.05) using the survival package in R. Subsequently, to enhance model generalizability and reduce overfitting, we performed LASSO regression analysis with the glmnet R package, standardizing all predictor variables prior to analysis. The optimal penalty parameter (lambda.min) was selected through 1000 iterations of cross-validation, identifying the value that yielded the minimum cross-validation error. The prognostic model was independently validated using the GSE53625 cohort from the GEO database. We developed distinct risk stratification models for both the training and validation cohorts, calculating each patient’s risk score as a linear combination of prognostic DE-ERG expression levels. This risk scoring system exhibited strong predictive performance, with higher scores significantly associated with poorer survival outcomes. The riskScore was calculated according to the formula:


$$\text{Riskscore}=\sum_{i=1}^{n}(mrnaexp_{i}\times coef_{i})$$

In the risk score calculation, “n” represents the total number of prognostically significant DE-ERGs in ESCC, while “i” denotes each individual gene among these. The regression coefficient for each gene is indicated by “coef”. For every patient, the riskScore was computed as a linear combination of the expression levels of these genes weighted by their respective coefficients. This score was calculated for each sample in both the training and validation cohorts. Using the median riskScore from the training cohort as a cutoff, patients were stratified into high- and low-risk groups. To maintain consistency, the same median cutoff value was applied to classify samples in the validation cohort into corresponding risk categories.

### 2.5 Validation of the risk prognostic model

During the initial analytical phase, we used R to generate comprehensive graphical representations—including risk score distribution curves, survival status distributions, and risk-associated heatmaps—for both the training and validation cohorts. These visualizations facilitated a systematic evaluation of survival in ESCC patients. Subsequently, using the survival and survminer packages in R, we conducted survival analysis to compare potential survival differences between risk groups. In addition, receiver operating characteristic (ROC) curve analysis was performed with the same packages to evaluate the predictive accuracy of the riskScore model and established clinical parameters. Finally, multivariate Cox regression analysis, implemented via the survival package in R, was applied to determine whether the riskScore retained independent prognostic value after adjusting for other clinical covariates.

### 2.6 Differential analysis of risk prognostic model

To systematically evaluate the expression patterns of the DE-ERGs included in our prognostic risk model, we conducted comparative analyses using the TCGA-ESCC dataset. First, we examined differences in DE-ERGs expression between tumor and normal tissues. Following data standardization, a heatmap was generated to visualize these expression profiles. We then performed subgroup analyses comparing DE-ERGs expression between high-risk and low-risk patients within both the training and validation cohorts. Differential expression patterns were visualized using box plots created with the “reshape2” and “ggpubr” packages in R, while hierarchical clustering heatmaps were constructed using the “pheatmap” package to comprehensively illustrate expression variations across risk groups and sample types.

### 2.7 Clinical features analysis

To evaluate the clinical applicability of our prognostic risk model, we performed comprehensive subgroup validation analyses in the training cohort using the survival and survminer packages in R. This stratified assessment enabled a rigorous evaluation of the model’s predictive performance across key clinicopathological variables, testing its robustness and generalizability across diverse patient populations. Specifically, we conducted stratified analyses based on gender (male vs. female), tumor invasion depth (T1–2 vs. T3–4), nodal status (N0 vs. N1–3), distant metastasis (M0 vs. M1), and overall tumor stage (I–II vs. III–IV).

### 2.8 Immune infiltration cell correlation analysis

To characterize the immune landscape of ESCC, we applied the CIBERSORT algorithm implemented with the e1071, parallel, and preprocessCore R packages to estimate the relative proportions of 22 distinct immune cell types based on TCGA transcriptomic profiles. Only samples meeting the significance threshold (P < 0.05) for immune cell fraction estimation were retained to ensure data reliability. We then performed comprehensive correlation analyses to examine: (1) associations between the DE-ERGs included in our prognostic model and immune cell infiltration patterns (using a significance threshold of P < 0.001), and (2) relationships between riskScore values in the training cohort and immune infiltration patterns (with a significance threshold of P < 0.05). Spearman correlation analysis served as the core statistical method. All analyses were conducted using the limma, reshape2, ggpubr, and ggExtra packages in R, facilitating a systematic evaluation of immune-microenvironment interactions in relation to both molecular and clinical prognostic features.

### 2.9 Single sample gene set enrichment analysis (ssGSEA)

To comprehensively evaluate the immune microenvironment characteristics in ESCC, we performed ssGSEA using the GSVA, limma, and GSEABase packages in R. This method enabled the quantification of enrichment scores for both immune cell subsets and immune-related functional pathways. Subsequently, we utilized the limma, reshape2, and ggpubr packages to conduct comparative analyses of immune profiles between high- and low-risk patient subgroups in the training cohort, systematically assessing differences in immune cell infiltration and functional activity.

### 2.10 Differential analysis of immune checkpoints

To systematically assess differential expression patterns of immune checkpoint-related genes across risk stratifications within the training cohort, we conducted comprehensive analyses utilizing the R environment. Employing the computational functionalities of the limma, reshape2, ggplot2, and ggpubr packages, we performed comparative evaluations to identify statistically significant variations in immune checkpoint gene expression profiles between high- and low-risk patient subgroups.

### 2.11 Drug sensitivity analysis

In order to identify potential therapeutic agents stratified according to prognostic risk within the training cohort, a systematic drug sensitivity analysis was performed using the limma, ggpubr, and pRRophetic packages in R. By applying a stringent statistical threshold (P < 0.001), this computational pharmacogenomic methodology facilitated the discernment of compounds demonstrating differential efficacy between high- and low-risk patient subgroups as defined by our prognostic model. The results uncovered clinically relevant pharmacological agents that could inform risk-stratified treatment strategies for ESCC patients, thereby advancing the framework for personalized therapeutic interventions.

### 2.12 Cell culture

This study utilized two human ESCC cell lines (KYSE-30 and KYSE-150), with normal esophageal epithelial cells (NE-1) serving as controls. The ESCC cell lines were cultured in RPMI 1640 medium supplemented with 10% fetal bovine serum (FBS), while normal esophageal epithelial cells were maintained in a mixed medium composed of Defined Keratinocyte-SFM (DK-SFM) and Epilife medium to preserve their epithelial characteristics. All cell lines were incubated at 37 °C in a humidified atmosphere of 5% CO_2_ to ensure optimal growth conditions.

### 2.13 Real-time quantitative PCR

Total RNA was extracted from ESCC cell lines (KYSE-30 and KYSE-150) and normal esophageal epithelial cells (NE-1) using TRIzol Reagent (Life Technologies Invitrogen; Cat. #15596018) following the manufacturer’s instructions. Complementary DNA (cDNA) was synthesized from equal amounts of RNA through reverse transcription, and subsequent amplification was carried out with ChamQ Universal SYBR qPCR Master Mix (Vazyme; Cat. #Q711-02). RT-qPCR was employed to evaluate mRNA expression levels of target DE-ERGs. The β-actin gene was used as an internal reference for normalization, and relative expression was calculated via the comparative threshold cycle (2^(-ΔΔCt)) method.

### 2.14 Statistical analysis

All statistical analyses and data visualizations were conducted using R statistical software (v4.1.2) and GraphPad Prism (v9.0.0). Differences between groups were assessed through one-way analysis of variance (ANOVA), with a significance threshold set at P < 0.05. To ensure methodological rigor and reproducibility, all experimental procedures were independently repeated in triplicate.

## 3 Results

### 3.1 DE-ERGs of ESCC

**Figure 2:**
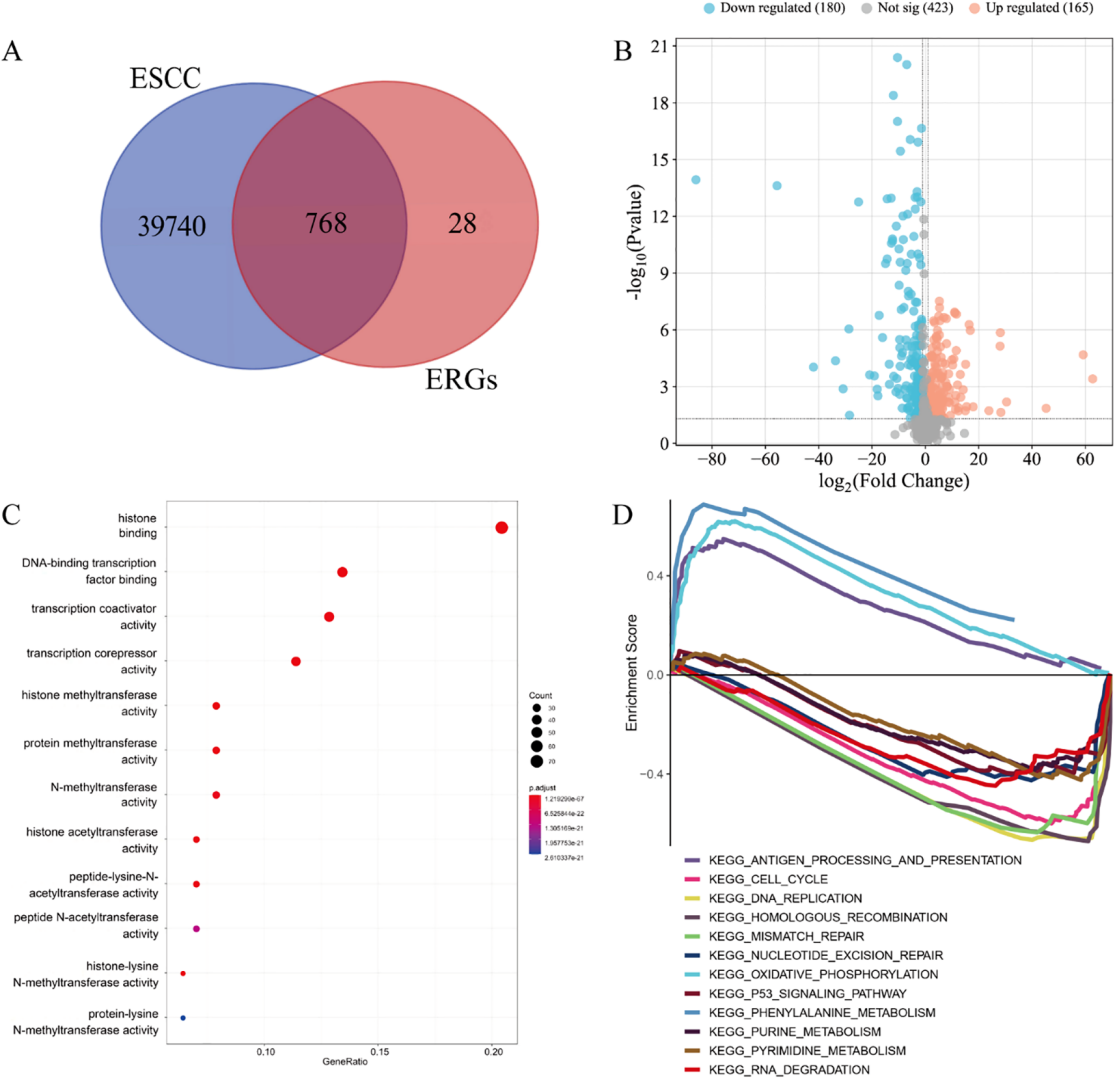
DE-ERGs of ESCC. **(A)** 768 ERGs potentially implicated in ESCC pathogenesis. **(B)** 345 DE-ERGs in ESCC. **(C)** GO enrichment analysis. **(D)** GSEA pathway enrichment analysis.

Through computational intersection of the TCGA-ESCC transcriptomic dataset with a curated set of 796 ERGs, we identified 768 ERGs potentially associated with ESCC pathogenesis (**Figure 2A**). Subsequent differential expression analysis of these candidate ERGs uncovered 345 DE-ERGs in ESCC relative to normal controls (**Figure 2B**).

### 3.2 Enrichment analysis

The GO enrichment of 345 DE-ERGs was mainly concentrated on: histone binding, DNA-binding transcription factor binding, transcription coactivator activity, transcription corepressor activity, histone methyltransferase activity, protein methyltransferase activity, N-methyltransferase activity, histone acetyltransferase activity, peptide-lysine-N-acetyltransferase activity, peptide N-acetyltransferase activity, histone-lysine N-methyltransferase activity and protein-lysine N-methyltransferase activity (**Figure 2C**). The patyway enrichment of 345 DE-ERGs was mainly concentrated on: antigen processing and presentation, cell cycle, DNA replication, homologous recombination, mismatch repair, nucleotide excision repair, oxidative phosphorylation, p53 signaling pathway, phenylalanine metabolism, purine metabolism, pyrimidine metabolism and RNA degradation (**Figure 2D**).

### 3.3 Construction of risk prognostic model

**Figure 3:**
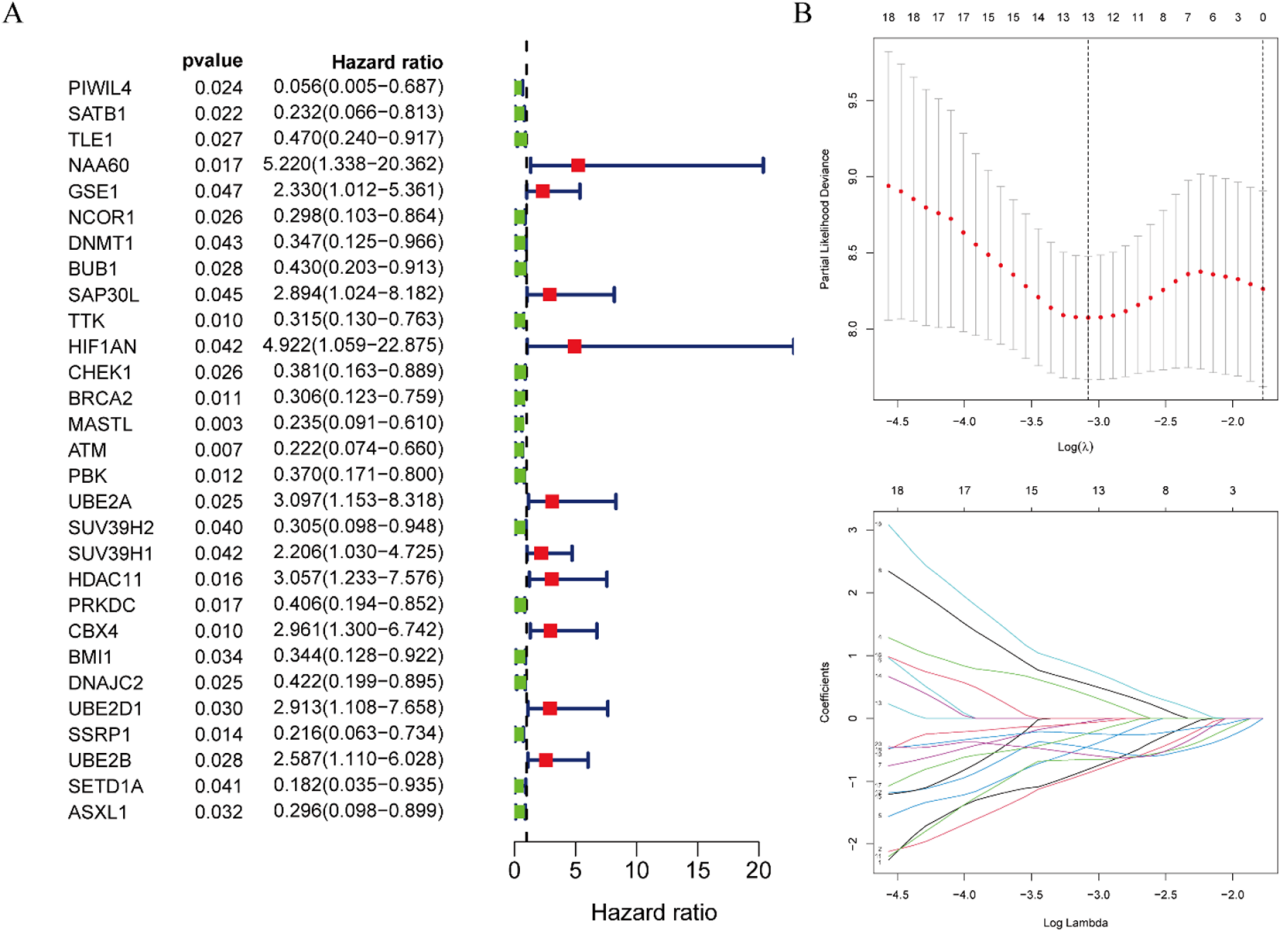
Construction of risk prognostic model. **(A)** Univariate Cox regression analysis obtained 29 candidates prognostic DE-ERGs. **(B)** LASSO regression analysis.

Univariate Cox proportional hazards regression analysis of the 345 DE-ERGs revealed 29 genes significantly associated with patient prognosis (P < 0.05) (**Figure 3A**). To enhance model generalizability and reduce overfitting, LASSO regression analysis was employed, identifying 13 optimal prognostic DE-ERGs based on minimal cross-validation error (**Figure 3B**). Individual risk scores were computed for all samples using the established risk score algorithm. In the training cohort (TCGA-ESCC), patients were categorized into high-risk (n = 40) and low-risk (n = 40) subgroups using the median riskScore as the cutoff. This stratification approach was subsequently validated in the external GEO cohort, which was similarly divided into high-risk (n = 90) and low-risk (n = 89) groups.

### 3.4 Validation of the risk prognostic model

**Figure 4:**
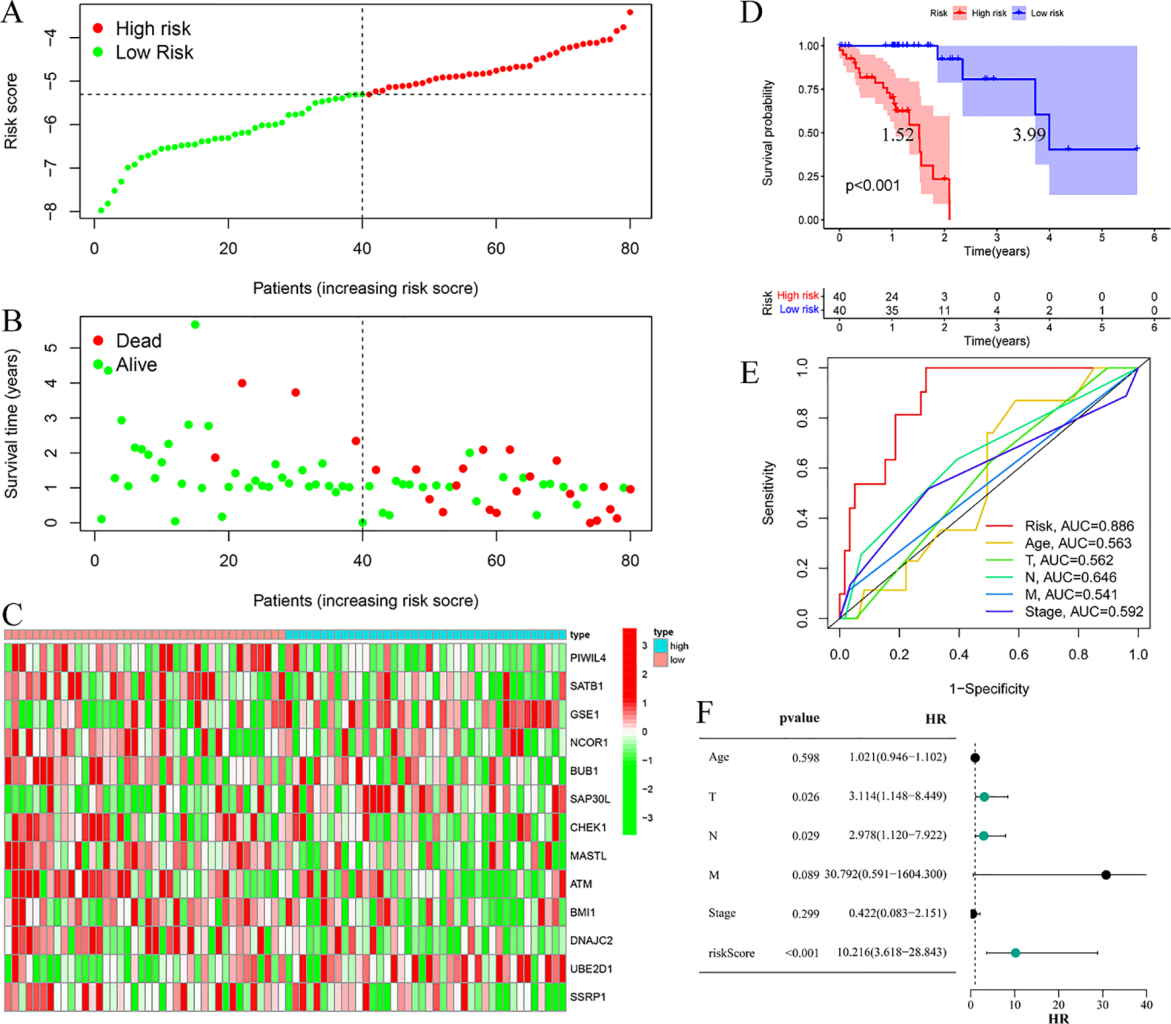
Training cohort. **(A)** Risk curve. **(B)** Survival status map; **(C)** Risk heatmap; **(D)** Survival curve, the figure highlights the median survival periods of the high-risk group and the low-risk group. **(E)** ROC curve; **(F)** Multivariate Cox regression analysis.

**Figure 5:**
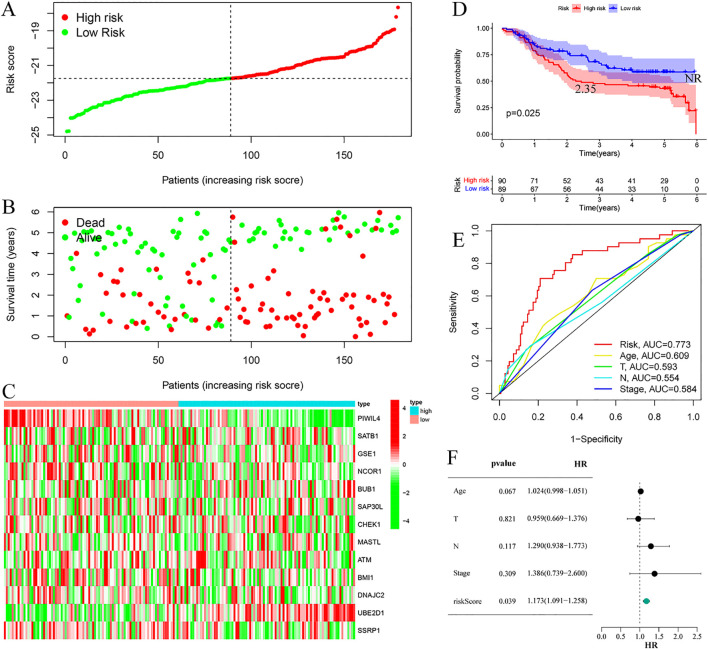
Validation cohort. **(A)** Risk curve. **(B)** Survival status map; **(C)** Risk heatmap; **(D)** Survival curve, the figure highlights the median survival periods of the high-risk group and the low-risk group, NR stands for “not reached”. **(E)** ROC curve; **(F)** Multivariate Cox regression analysis.

Risk stratification analysis revealed a consistent positive association between risk scores and disease progression in both cohorts, with high-risk patients demonstrating significantly elevated ESCC risk relative to low-risk individuals (**Figures 4A**, **5A**). Mortality analysis further supported these observations, indicating markedly higher fatality rates in the high-risk subgroups (**Figures 4B**, **5B**). Expression heatmaps of the 13 prognostic ERGs displayed distinct molecular signatures differentiating the risk categories (**Figures 4C**, **5C**). Survival analysis confirmed significantly worse clinical outcomes among high-risk patients in both the training and validation cohorts (**Figures 4D**, **5D**). ROC curve evaluation indicated that the riskScore exhibited superior predictive performance compared to conventional clinical parameters, with nodal stage (N stage) and age identified as additional significant prognostic indicators in the training and validation cohorts, respectively (**Figures 4E**, **5E**). Multivariate Cox regression analyses performed across both cohorts confirmed that the riskScore remained an independent prognostic factor for ESCC after adjustment for relevant clinical covariates (**Figures 4F**, **5F**).

### 3.5 Differential analysis of risk prognostic model

**Figure 6:**
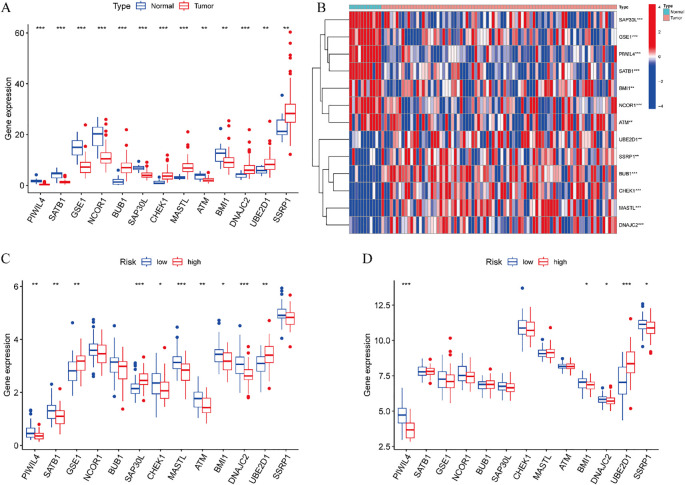
Differential analysis of risk prognostic model. **(A)** Expression profiles of 13 DE-ERGs. **(B)** Difference expression heatmap of 13 DE-ERGs. **(C)** The differences of DE-ERGs in the training cohort. **(D)** The differences of DE-ERGs in the validation cohort. Gene expression levels are shown as log2(TPM + 1) values derived from RNA-seq analysis. Statistical significance between groups was determined by the Wilcoxon rank-sum test. *p < 0.05, **p < 0.01, ***p < 0.001.

Comprehensive expression profiling of the 13 DE-ERGs included in our prognostic model revealed distinct patterns in ESCC. Specifically, PIWIL4, SATB1, GSE1, NCOR1, SAP30L, ATM, and BMI1 were significantly downregulated in tumor tissues relative to normal controls, while BUB1, CHEK1, MASTL, DNAJC2, UBE2D1, and SSRP1 showed pronounced upregulation (**Figures 6A, B**). Comparative analysis between high- and low-risk groups within the training cohort indicated significant differential expression of PIWIL4, SATB1, GSE1, SAP30L, CHEK1, MASTL, ATM, BMI1, DNAJC2, and UBE2D1 (**Figure 6C**). This expression signature was partially conserved in the validation cohort, with PIWIL4, BMI1, DNAJC2, UBE2D1, and SSRP1 demonstrating consistent risk-associated dysregulation (**Figure 6D**). These results suggest that the identified DE-ERGs may contribute critically to ESCC progression and facilitate molecular risk stratification.

### 3.6 Clinical features analysis

**Figure 7:**
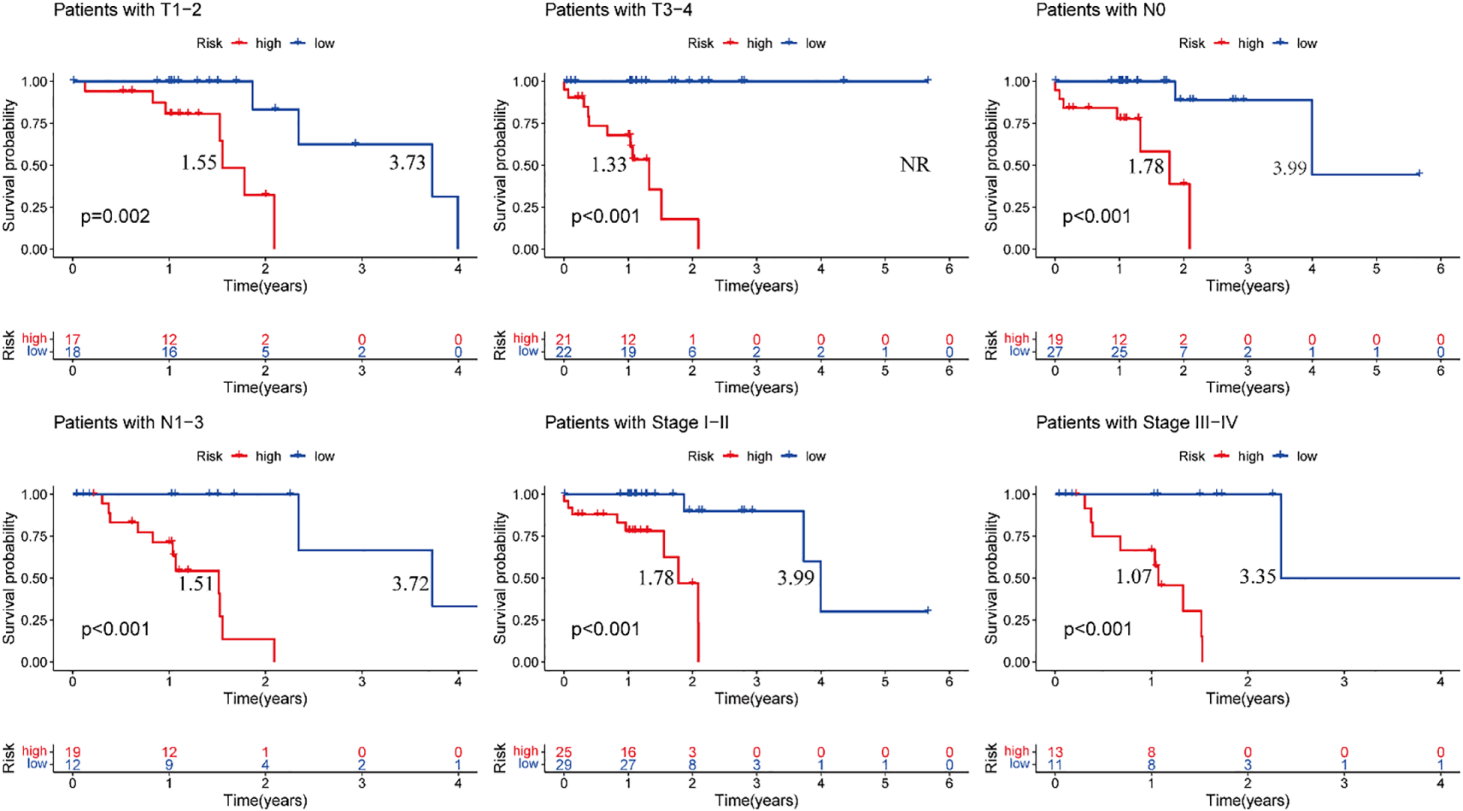
Training cohort, clinical validation of the risk prognosis model. The figure highlights the median survival periods of the high-risk group and the low-risk group. NR stands for “not reached”.

Stratified survival analysis confirmed the robust prognostic capacity of our risk model across major clinicopathological variables. The model consistently discriminated survival outcomes between T1–2 and T3–4 tumor stages, nodal involvement status (N0 vs N1–3), and overall disease stage (I–II vs III–IV) (**Figure 7**). These findings highlight the clinical applicability of our prognostic signature in diverse patient populations with heterogeneous disease features.

### 3.7 Immune infiltration cell correlation analysis

**Figure 8:**
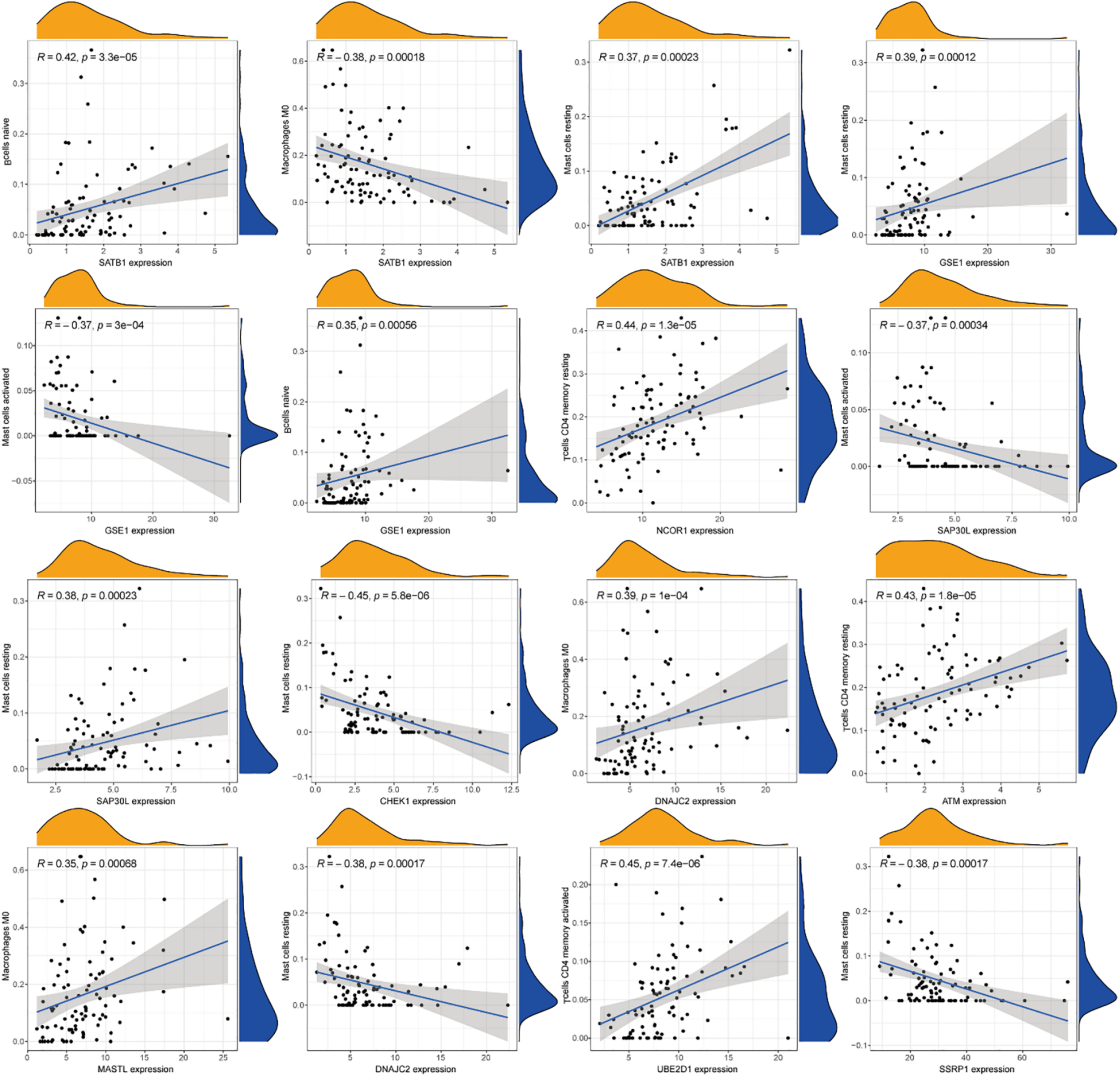
Comprehensive correlation analysis between the 13 DE-ERGs and tumor-infiltrating immune cells.

**Figure 9:**
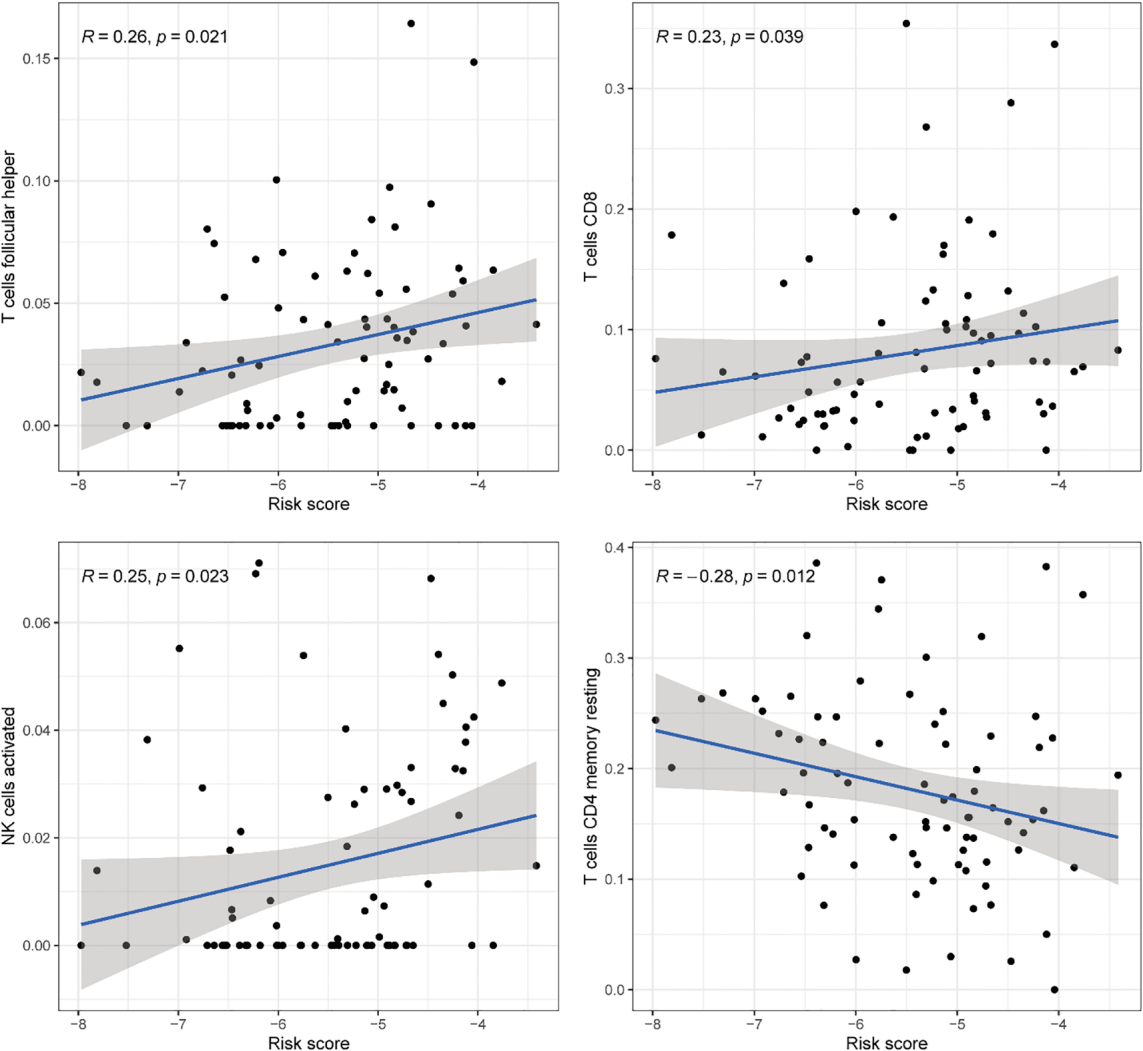
Comprehensive correlation analysis between the risk prognostic model of the training cohort and tumor-infiltrating immune cells.

Comprehensive correlation analysis between the 13 DE-ERGs and tumor-infiltrating immune cells revealed distinct immunomodulatory associations. Specifically, SATB1 was positively correlated with naive B cells and resting mast cells, but negatively correlated with macrophages M0. GSE1 is positively correlated with naive B cells and resting mast cells, but negatively correlated with activated mast cells. NCOR1 is positively correlated with CD4 memory resting T cells. SAP30L is positively correlated with resting mast cells, but negatively correlated with activated mast cells. CHEK1 is negatively correlated with resting mast cells. DNAJC2 is positively correlated with M0 macrophages and negatively correlated with resting mast cells. ATM is positively correlated with the CD4 memory resting state of T cells. MASTL is positively correlated with macrophages M0. UBE2D1 is positively correlated with T cells CD4 memory activated. SSRP1 is negatively correlated with mast cells resting (**Figure 8**). The correlation analysis between immune infiltrating cells and the riskScore of the risk prognostic model revealed that: the riskScore was positively correlated with activated NK cells, T cells CD8, and T cells follicular helper, while negatively correlated with T cells CD4 memory resting (**Figure 9**). These results suggest that specific expression patterns of DE-ERGs may influence the tumor immune microenvironment through differential regulation of immune cell infiltration.

### 3.8 ssGSEA

**Figure 10:**
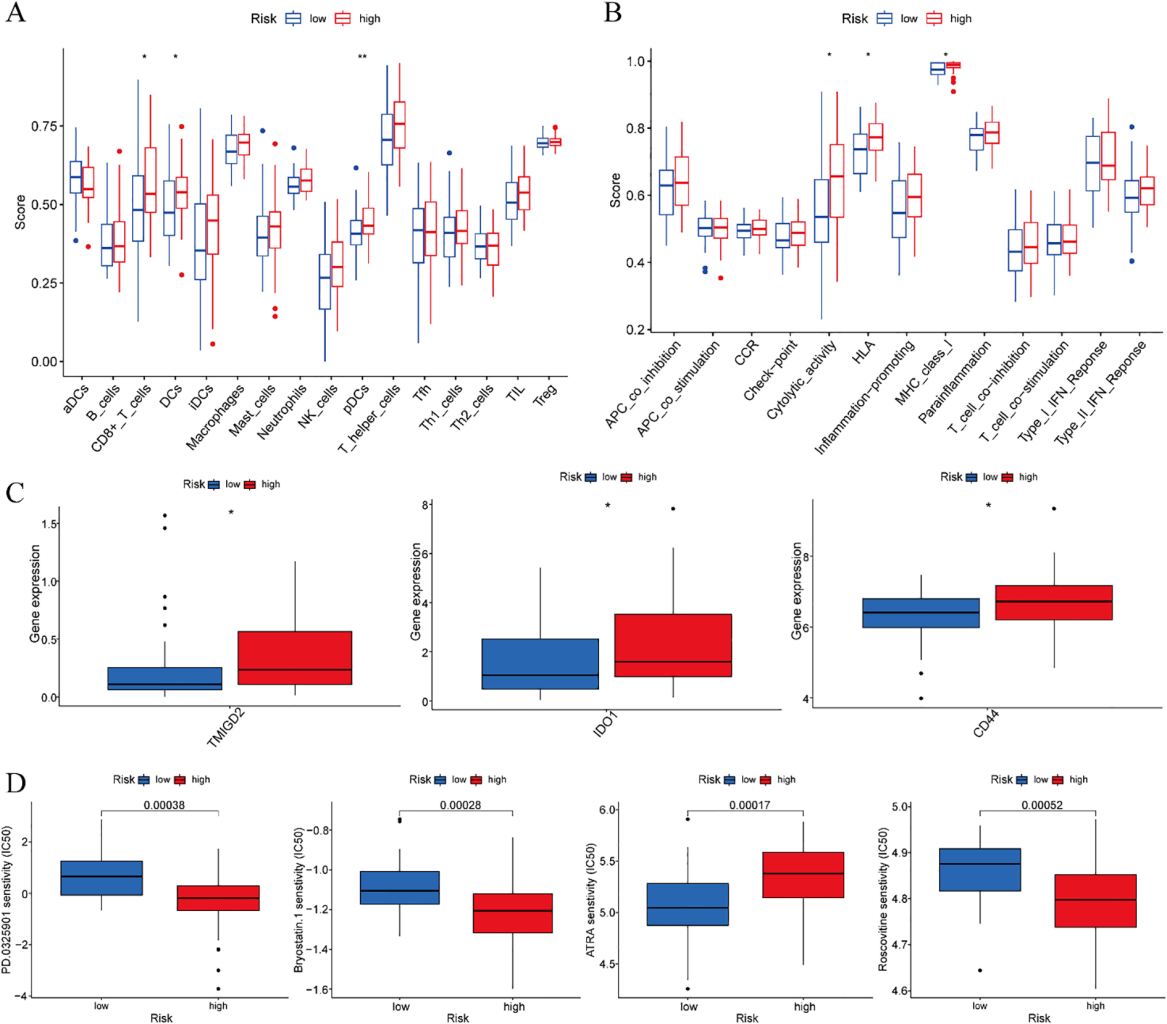
Training cohort. **(A)** Analysis of differences in immune cells. **(B)** Analysis of differences in immune function. **(C)** Differential analysis of immune checkpoints. **(D)** Drug sensitivity analysis. *p < 0.05, **p < 0.01.

Comprehensive immune characterization of the risk-stratified cohorts revealed significant enrichment of CD8^+^ T cells, dendritic cells (DCs), and plasmacytoid dendritic cells (pDCs) in high-risk ESCC patients relative to low-risk individuals (**Figure 10A**). Furthermore, functional analysis indicated elevated levels of cytolytic activity, HLA expression, and MHC class I activity in the high-risk subgroup (**Figure 10B**).

### 3.9 Differential analysis of immune checkpoints

Comparative analysis of immune checkpoint molecules revealed significant differential expression of three key immunoregulatory genes—TMIGD2, IDO1, and CD44—between risk-stratified groups within the training cohort. Each of these molecules exhibited distinct expression profiles that clearly distinguished high-risk from low-risk ESCC patients (**Figure 10C**). These results suggest that immune checkpoint regulation may be altered during disease progression, with potential implications for differential therapeutic responses across prognostic subgroups.

### 3.10 Drug sensitivity analysis

Systematic drug sensitivity analysis identified four compounds demonstrating differential efficacy between the prognostic risk groups. Specifically, low-risk patients exhibited increased sensitivity to PD-0325901, Bryostatin-1, and Roscovitine, whereas high-risk patients displayed greater responsiveness to ATRA (**Figure 10D**). These results suggest distinct molecular vulnerabilities among risk-stratified ESCC subtypes, which may guide the development of personalized therapeutic approaches.

### 3.11 Validation of ERGs expression in ESCC

**Figure 11:**
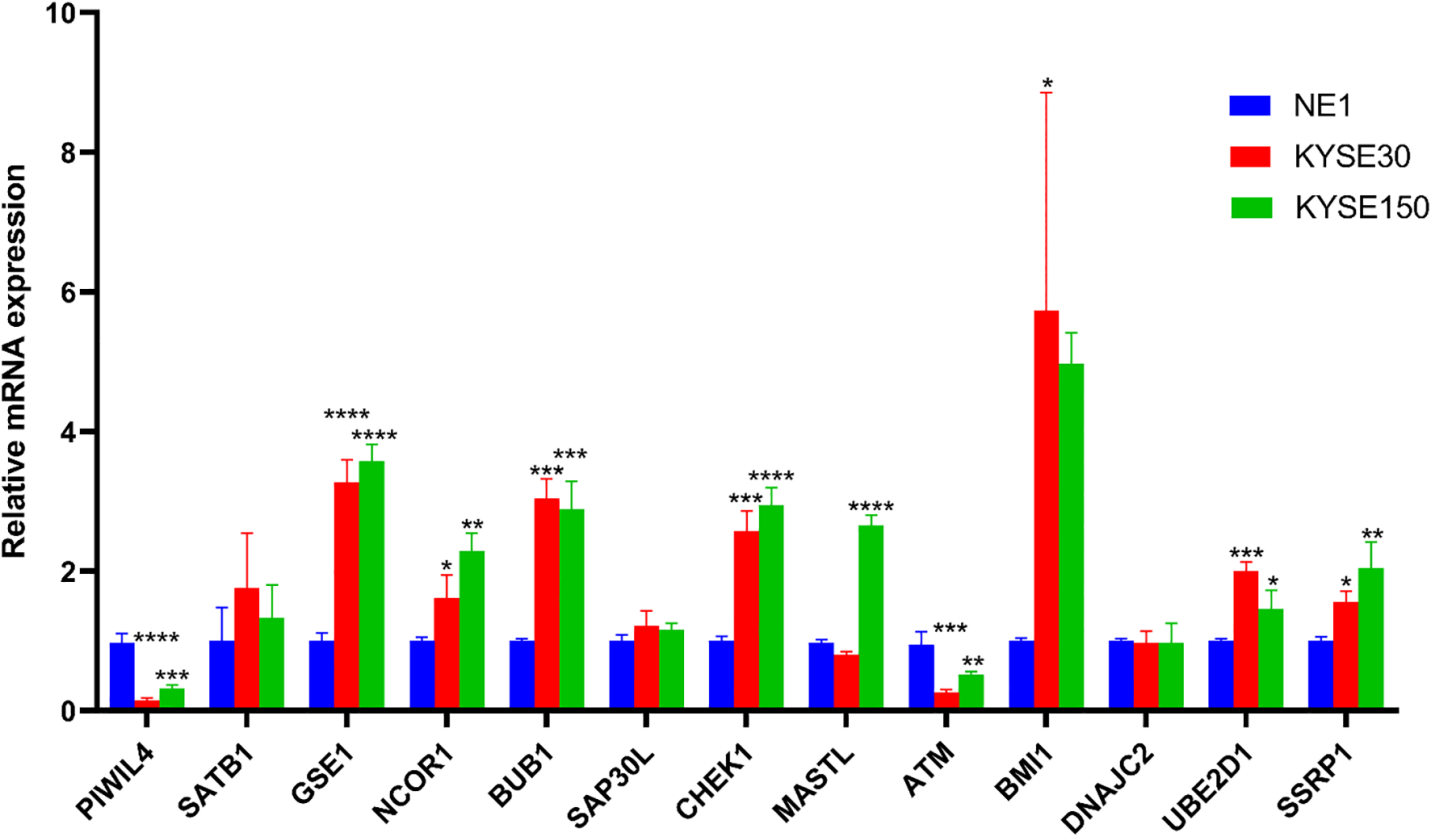
Validation of the mRNA expression level of DE-ERGs in ECCC cell lines. *p < 0.05, **p < 0.01, ***p < 0.001, each experiment was repeated three times.

All primer sequences, detailed in **Table 1**, were synthesized by Accurate Biology. RT-qPCR analysis using validated primers revealed distinct dysregulation patterns of the DE-ERGs in ESCC cell lines relative to normal esophageal epithelial cells (NE-1). Significant upregulation of GSE1, NCOR1, BUB1, CHEK1, UBE2D1, and SSRP1 was observed in both KYSE-30 and KYSE-150 cell lines, while MASTL overexpression was specific to KYSE-150 and BMI1 to KYSE-30. In contrast, PIWIL4 and ATM showed consistent downregulation across both cell lines (**Figure 11**). These results validate the prognostic DE-ERG signature and underscore cell line-specific epigenetic alterations implicated in ESCC pathogenesis.

**Table 1:** List of primers.

Gene	Forward	Reverse
PIWIL4	ATACCAGCTCAAGACTGTCGG	CATACCATTCGTTACGTGTTGCT
SATB1	TGCCAATCCTCCTCTTGTTACCTG	GCACAAAACGCTATGTCATGCC
GSE1	TGTGTTGCCATGTTACTATGCC	TACTGACAATGCACCCAACCT
NCOR1	AGCTCCATCCTCTCCAATTTCG	TAGCTGCCTCTTCTTCAAGCTG
BUB1	AACTTGCGTCTACACCATTCCAC	TGGGCTTTTCTCTTGAATTGGACT
SAP30L	ACATTCTGCCTACAACCATCCCA	TACAAAGAACAGGCTTCTCCACGA
CHEK1	CTCAGACTTTGGCTTGGCAAC	TTCTCCAGCGAGCATTGCAGT
MASTL	CCCAAATCAGATCAAGTCGGGAA	GCCCTGCCTAGTAACAGCTC
ATM	ACTATCCCAATACACTGCTGGAGA	TTTGAGCAACTGACTGGCAAAC
BMI1	TAGTATGAGAGGCAGAGATCGGG	TTTATTCTGCGGGGCTGGGAG
DNAJC2	CATGCTGAAAACACTTGATCCCA	TGATCTGTCTCTGTGTAGCCTT
UBE2D1	GAGTGATCTACAGCGCGATCC	GGCCCCATAATAGTGGCTTGC
SSRP1	GCCTGAGGAGATTCCCAACCT	GGCTGCACAAGGGAAACCAA
β-actin	TGGCACCCAGCACAATGAA	CTAAGTCATAGTCCGCCTAGAAGCA

## 4 Discussion

In this study, we developed a novel ERGs-based prognostic model that exhibits robust predictive performance for survival outcomes in ESCC patients and offers mechanistic insights into immune microenvironment regulation. The refined signature consists of 13 DE-ERGs—PIWIL4, SATB1, GSE1, NCOR1, BUB1, SAP30L, CHEK1, MASTL, ATM, BMI1, DNAJC2, UBE2D1, and SSRP1—each showing significant dysregulation in ESCC pathogenesis. Comprehensive immune characterization revealed a distinct immunophenotype in high-risk patients, marked by increased infiltration of CD8^+^ T cells, DCs, and pDCs, along with elevated cytolytic activity, HLA expression, and MHC class I activity. We further identified three immune checkpoint molecules—TMIGD2, IDO1, and CD44—with expression levels correlated to risk stratification. Pharmacogenomic evaluation highlighted four potential therapeutic compounds—PD-0325901, Bryostatin-1, ATRA, and Roscovitine—demonstrating differential efficacy between risk subgroups. Finally, experimental validation using RT-qPCR confirmed the expression patterns of all 13 prognostic DE-ERGs in ESCC cell lines.

Based on their core biological functions, the 13 DE-ERGs can be classified into four principal functional categories. The first group comprises factors involved in chromatin remodeling and transcriptional regulation—SATB1, NCOR1, SAP30L, BMI1, and SSRP1—which mediate gene silencing or activation through higher-order chromatin organization and recruitment of histone-modifying complexes (17–21). SATB1 encodes a nuclear matrix attachment region-binding protein that orchestrates chromatin architecture by tethering genomic loci to the nuclear scaffold, recruiting chromatin-remodeling complexes to dynamically regulate transcription. Emerging evidence indicates that Wnt/β-catenin signaling upregulates SATB1 to drive colorectal cancer initiation and progression (22), while PAK5-mediated phosphorylation enhances its oncogenic potential in cervical cancer (23). Additionally, reversible ubiquitination of SATB1 by USP47 and SMURF2 promotes colon cancer proliferation (24). In ESCC, triptolide exerts anti-tumor effects via the circNOX4/miR-153-3p/SATB1 axis (25). NCOR1, a transcriptional corepressor, mediates ligand-independent repression of nuclear receptors through chromatin condensation and transcription factor exclusion. It serves as an independent prognostic marker in breast cancer (26), disrupts PPARα/γ signaling in prostate cancer (27), and promotes proliferation and senescence resistance in colorectal cancer (28). In HPV-associated cervical cancer, the E6 protein recruits NCOR1 to facilitate OCT4-mediated p53 suppression (29). SAP30L, a component of histone deacetylase complexes, represses RNA polymerase II-mediated transcription. The long non-coding RNA SAP30L-AS1 promotes prostate cancer progression by epigenetically silencing SAP30L (30). BMI1, a core subunit of polycomb repressive complex 1, acts as an oncogenic stem cell regulator and is frequently dysregulated in cancers. In ESCC, miR-218 suppresses tumor growth by targeting BMI1 (31), which serves as both a cancer stem cell marker and therapeutic target (32). Chlorogenic acid exhibits anti-tumor activity in ESCC through dual inhibition of BMI1 and SOX2 (33). SSRP1, a subunit of the FACT complex, facilitates transcriptional elongation and DNA damage response and demonstrates oncogenic properties in multiple malignancies. It regulates tumor growth and apoptotic resistance via AKT signaling in colorectal cancer (34), and co-overexpression with APE1 correlates with aggressive phenotypes and poor prognosis in bladder cancer (35).

The second category encompasses genes involved in cell cycle checkpoint control and genome integrity maintenance—BUB1, CHEK1, MASTL, and ATM—which function as key components of the spindle assembly checkpoint and DNA damage response pathways (36–39). BUB1, a critical mitotic serine/threonine kinase, regulates chromosome segregation and contributes to DNA damage response. In bladder cancer, it promotes oncogenesis through STAT3 pathway activation (40), and in triple-negative breast cancer, it confers radioresistance via regulation of non-homologous end joining (41). BUB1 expression also shows promise as a predictive biomarker for immunotherapy response and clinical outcomes in breast cancer (42). CHEK1, a Ser/Thr protein kinase, plays a central role in DNA damage checkpoint control by inducing cell cycle arrest in response to genomic instability. Ginsenoside Ro has been shown to enhance 5-fluorouracil sensitivity in esophageal cancer by disrupting autophagic flux through the ESR2-NCF1-ROS pathway, leading to CHEK1-mediated DNA damage activation (43). Clinically, CHEK1 genetic polymorphisms are associated with postoperative prognosis in thoracic ESCC patients after radical resection (44). MASTL, a microtubule-associated serine/threonine kinase initially linked to autosomal dominant thrombocytopenia, contributes to oncogenesis through multiple mechanisms. It promotes chromosomal instability and metastasis in breast cancer (45), enhances tumor progression and chemoresistance via Wnt/β-catenin signaling in colorectal cancer (46), and modulates EGFR signaling in pancreatic cancer (47). ATM, a PI3K-related kinase, acts as a master regulator of DNA damage response alongside ATR to maintain genomic integrity. In ESCC, the long noncoding RNA SNHG20 drives tumor progression by activating the ATM-JAK-PD-L1 axis (48). HMGB1-mediated radioresistance in ESCC involves PI3K/AKT/ATM pathway activation (49), and ATM polymorphisms may serve as predictors of radiation therapy outcomes (50).

The third functional category involves the regulation of pluripotency and cell fate determination, represented by PIWIL4. Genes in this group support stem cell self-renewal and pluripotency, and their dysregulation is often associated with abnormal cellular reprogramming and dedifferentiation (51). PIWIL4, a member of the evolutionarily conserved Argonaute protein family, is essential for germline stem cell maintenance and development. Clinically, reduced expression of PIWIL4, along with PIWIL1 and PIWIL2, is correlated with unfavorable survival in renal cell carcinoma (52). In breast cancer, PIWIL4 shows marked overexpression in primary tumors and the MDA-MB-231 cell line. Functional analyses indicate that PIWIL4 knockdown significantly inhibits cell migration and induces apoptosis, with only minimal effects on proliferation (53). Furthermore, the PIWIL4/SUPT5H complex has been identified as a promising prognostic biomarker for predicting clinical outcomes and immune microenvironment features in intrahepatic cholangiocarcinoma (54).

The fourth category comprises multifunctional auxiliary regulators—GSE1, DNAJC2, and UBE2D1—which play essential cooperative roles across diverse biological processes. GSE1 encodes a proline-rich nuclear protein with coiled-coil domains that functions as part of the BRAF35-HDAC repressor complex. This oncogenic driver is overexpressed in breast cancer, where its activity is suppressed by the tumor-suppressive miR-489-5p (55). In gastric cancer, GSE1 promotes tumor progression via SLC7A5-mediated enhancement of growth and metastasis (56), and contributes to trastuzumab resistance (57). DNAJC2, a member of the M-phase phosphoprotein family frequently altered in head and neck squamous cell carcinomas, acts as an oncogenic driver in colorectal cancer. Its expression is negatively regulated by miR-627-3p, and overexpression accelerates uncontrolled proliferation (58). UBE2D1, an E2 ubiquitin-conjugating enzyme, mediates ubiquitination of p53 and HIF1α through E1-E3 interactions and plays a significant role in gastric cancer pathogenesis. Knockdown of UBE2D1 impairs cancer cell migration by reducing SMAD4 ubiquitination (59).

The prognostic model established in this study integrates 13 ERGs with demonstrated predictive utility in ESCC. Among these, four genes—SATB1, CHEK1, ATM, and BMI1—have previously documented roles in ESCC pathogenesis. In contrast, the remaining nine genes (PIWIL4, GSE1, NCOR1, BUB1, SAP30L, MASTL, DNAJC2, UBE2D1, and SSRP1) represent novel contributors, as their potential functions in ESCC progression and prognosis had not been previously elucidated. Our comprehensive analysis indicates that these nine genes not only exhibit strong prognostic biomarker potential but are also significantly involved in modulating the ESCC immune microenvironment. These findings offer valuable insights that may inform future research into epigenetic mechanisms underlying ESCC tumor biology, immune regulation, and therapeutic resistance.

Accumulating evidence highlights the critical role of epigenetic dysregulation in shaping tumor biology and influencing therapeutic responses. In cancer cells, an altered epigenome remodels the immune landscape of the tumor microenvironment (TME), undermining antitumor immunity, accelerating malignant progression, and promoting resistance to immunotherapy. Key epigenetic changes—such as abnormal histone post-translational modifications, DNA methylation patterns, and RNA modifications—distinguish malignant from nonmalignant cells and regulate oncogene and tumor suppressor function, thereby driving tumorigenesis. Single-cell transcriptomic and epigenomic analyses have revealed associations between chromatin accessibility states and immune cell composition within tumors. Epigenetic plasticity in cancer is closely tied to genes located in open chromatin regions that enable intercellular communication. Moreover, epigenetic enzymes and transcriptional regulators in malignant cells control the expression of ligands, receptors, and cytokines essential for immune cell differentiation, migration, and activation (60). Therapeutic targeting of epigenetic machinery offers potential for reprogramming the TME through transcriptional and metabolic changes in local immune populations. Such approaches may inhibit immunosuppressive cells (e.g., MDSCs and Tregs) while promoting the function of antitumor effector T cells, professional antigen-presenting cells (APCs), and even cancer cells acting as nonprofessional APCs. Epigenetic modulators can also enhance tumor immunogenicity by reactivating silenced tumor-associated antigens, upregulating neoantigen expression and MHC machinery, and inducing immunogenic cell death (ICD) (61). Notably, epigenetic mechanisms contribute to immunotherapy resistance by modulating specific immune subsets within the TME (60). Therefore, combining epigenetic agents with immunotherapies represents an emerging strategic approach in oncology. Advances in the specificity and affinity of epigenetic drugs, along with the development of small molecules targeting a wider range of epigenetic and immune pathways—integrated with state-of-the-art genomic and immunomonitoring technologies—are expected to drive rational combination strategies and expand mechanistic understanding (62).

The ERGs identified in the present study were found to be significantly associated with immune-related processes. Specifically, the deficiency of SATB1 has been implicated in the initiation and progression of autoimmune disorders. In murine models, conditional knockout of Satb1 in CD4+ T cells resulted in T cell hyperactivation and widespread inflammatory cell infiltration across multiple organs. SATB1 appears to confer protection against immune-mediated tissue damage by modulating chemokine expression (63). In prostate cancer, GSE1 is frequently upregulated, whereas TACSTD2 exhibits downregulation; this inverse correlation promotes metastatic dissemination, castration resistance, and disease progression, while also modulating clinical and immune parameters in patients (64). Furthermore, NCOR1 plays an essential role in T cell development through its regulation of thymocyte survival. Additionally, NCOR1 fine-tunes the balance between immune tolerance and inflammation by controlling metabolic pathways such as glycolysis and fatty acid oxidation in dendritic cells across both murine and human models (65, 66). SAP30L demonstrates a positive correlation with resting mast cells and a negative association with activated mast cells, suggesting a modulatory role in mast cell function. In soft tissue sarcomas, CHEK1 serves as an unfavorable prognostic biomarker associated with immunosuppressive phenotypes, showing significant overexpression in immune-low tumors and correlating with altered patterns of tumor-infiltrating immune cells (67). Integrin-αvβ3 is upregulated on therapy-resistant tumor cells via chronic activation of ATM/Chk2 and NF-κB pathways. Inhibition of integrin-αvβ3 enhanced therapeutic responses by stimulating host immunity, mechanistically through impairing dendritic cell phagocytosis and subsequent T cell cross-priming (68). Tumor-infiltrating immune cells have emerged as critical determinants of immunotherapy efficacy. Prognostic models incorporating immune features—such as a ceRNA network involving MASTL, or populations including CD4+ memory T cells, monocytes, and neutrophils—show utility in predicting clinical outcomes in gastric cancer (69). UBE2D1, a gene linked to cuproptosis, serves as a prognostic indicator in lung adenocarcinoma and participates in shaping the immune microenvironment (70). Moreover, the SSRP1/SLC3A2 axis in arginine transport represents a novel therapeutic target to counteract immune evasion and tumor progression in peripheral T-cell lymphoma (71). Collectively, these findings suggest that the ERGs identified in this study may influence ESCC prognosis via regulation of the tumor immune microenvironment. Further mechanistic investigations are warranted to elucidate the precise underlying pathways.

IDO1 functions as a pleiotropic mediator involved in multiple pathophysiological processes, including antimicrobial defense, immunoregulation, neuropathology, and antioxidant responses. Predominantly expressed in antigen-presenting cells—such as dendritic cells, monocytes, and macrophages—IDO1 induces immunosuppression through tryptophan depletion leading to T cell anergy and the production of immunomodulatory kynurenine metabolites. Clinically, elevated IDO1 expression following neoadjuvant therapy is associated with poor pathologic response and unfavorable prognosis in ESCC (72). Moreover, tumor-associated IDO1 overexpression serves as an independent predictor of disease recurrence and distant metastasis (73). Integrated multi-omics analyses have identified IDO1 as a co-expression partner of PD-1 on tumor-associated macrophages, underscoring its utility as both a prognostic biomarker and a promising immunotherapeutic target in ESCC (74). CD44 facilitates cell-cell interactions, adhesion, and migration via extracellular matrix binding and growth factor receptor signaling. This multifunctional molecule contributes to lymphocyte homing, hematopoietic differentiation, and metastatic spread. In ESCC, microRNA-34a suppresses tumor progression by directly targeting CD44, thereby inhibiting invasion and metastasis (75). The TWIST1-CD44-MMP13 axis has been implicated in epithelial-mesenchymal transition, functioning as both a diagnostic marker and a therapeutic target in aggressive ESCC (76). TMIGD2, an immunoregulatory surface receptor, modulates T cell activation, angiogenesis, and cytokine production through coreceptor signaling. Growing evidence supports its clinical relevance across malignancies: microRNA-486-3p-mediated regulation of TMIGD2 influences cisplatin resistance in ovarian cancer (77), while miR-615-5p exerts antitumor effects in cervical cancer via TMIGD2 targeting (78).

PD-0325901 exhibits synergistic antitumor effects when combined with the CK2 inhibitor CX-4945 in head and neck squamous cell carcinoma, effectively countering therapeutic resistance (79). It has demonstrated promising clinical activity in phase I/II trials across multiple malignancies, including non-small cell lung cancer, advanced melanoma, hormone receptor-positive breast cancer, and KRAS-mutant colorectal and pancreatic cancers (80–83). Bryostatin-1, a macrocyclic lactone PKC modulator, has advanced to phase II clinical trials in various solid tumors, where it has shown disease-stabilizing properties (84). Mechanistically, it confers cytoprotection in prostate cancer by regulating PKC isoform translocation and inhibiting PKC-dependent TNF-α release (85). In advanced EC, sequential administration of Bryostatin-1 with paclitaxel has produced clinically meaningful antitumor responses (86). ATRA, the biologically active metabolite of vitamin A, serves as a key regulator of cellular differentiation and apoptosis through mechanisms involving nuclear receptor activation and epigenetic reprogramming (87). Beyond its established efficacy in acute promyelocytic leukemia and neuroblastoma, ATRA exhibits multifaceted antitumor effects, such as reprogramming pancreatic stellate cells to inhibit desmoplasia and invasion (88), suppressing colorectal carcinogenesis via miR-3666 (89), and reversing tamoxifen resistance in breast cancer through Pin1 targeting (90). Roscovitine demonstrates broad-spectrum anticancer activity via multiple mechanisms: inhibition of estrogen receptor-α phosphorylation in hormone-responsive breast cancer (91), cdk5-mediated regulation of invasive breast cancer proliferation (92), chemosensitization of colorectal cancer cells to conventional cytotoxic agents (93), and central analgesic effects through modulation of NMDA receptor 2B subunit expression (94). Although Bryostatin-1 has documented efficacy in EC, the other three agents—PD-0325901, ATRA, and Roscovitine—identified through our ESCC risk model represent novel therapeutic candidates worthy of further exploration in esophageal squamous cell carcinoma. Their established mechanisms across diverse cancers, coupled with our risk-stratified sensitivity results, position these compounds as promising candidates for targeted therapy development in ESCC.

In the present study, we systematically constructed and validated a comprehensive risk prediction model for ESCC based on ERGs using transcriptomic data from the TCGA database. The robustness and generalizability of this prognostic model were further confirmed through rigorous external validation with independent datasets from the GEO repository. Our results provide compelling evidence supporting the significant influence of this ERGs -based risk stratification system on both clinical outcomes and immune microenvironment features in ESCC patients. Additionally, experimental validation of key ERGs incorporated in the risk model was conducted via RT-qPCR. Despite these insights, several limitations should be acknowledged. First, the statistical power of our conclusions may be constrained by the relatively limited sample size in the current analysis; future multicenter studies with larger cohorts are warranted to further validate and refine the predictive model. Second, the precise biological functions, immunomodulatory roles, and molecular mechanisms of the identified ERGs in ESCC pathogenesis remain incompletely understood. Elucidating these aspects through comprehensive functional studies represents an essential direction for future research, which would not only deepen our understanding of ESCC biology but may also contribute to the development of novel epigenetically targeted therapeutic strategies.

## 5 Conclusion

This study systematically established and validated a robust 13-gene signature of DE-ERGs with significant prognostic value in ESCC. The model offers important insights into the interplay between epigenetic dysregulation and ESCC pathogenesis while improving predictive accuracy for clinical outcomes. Furthermore, comprehensive analyses revealed distinct immune microenvironment features linked to risk stratification, underscoring the potential of this signature to inform immunotherapeutic strategies. Integrated pharmacogenomic profiling identified four promising therapeutic agents showing differential sensitivity across risk subgroups. These findings open new avenues for targeted therapy development in ESCC. The present work constitutes a notable advancement in precision oncology for this malignancy, with meaningful implications for prognostic evaluation and personalized treatment. Future validation efforts and clinical translation of these results may substantially enhance therapeutic decision-making and improve outcomes in ESCC management.

## Data Availability

Publicly available datasets were analyzed in this study. This data can be found here: https://portal.gdc.cancer.gov/.
